# Closing the Gap to Interventions for Tuberous Sclerosis Complex–Associated Neuropsychiatric Disorders (TAND): Protocol for a Longitudinal Study of TAND Severity, Predictors, and Caregiver Well-Being (TANDem-2)

**DOI:** 10.2196/91726

**Published:** 2026-05-05

**Authors:** Petrus J de Vries, Nola Chambers, Erin Campbell, Lucas Gutierrez-Lafrentz, Tosca-Marie Heunis, Liezl Schlebusch, Guillaume Beaure d'Augères, Stacey Bissell, Anna Byars, Jamie Capal, Laís Cardozo, Sebastian Cukier, Peter Davis, Naomi Beth Epstein, Carla Fladrowski, Jennifer Flinn, Tanjala Gipson, Sarah Louise Goy, John Christopher Kingswood, Darcy Krueger, Francesca Little, Sugnet Lubbe, Rebecca Mitchell, Micaela Rozenberg, Mustafa Sahin, Mitchell Silva, Catherine Smith, Shoba Srivastava, Megumi Takei, Agnies van Eeghen, Mary Vasseghi, Jan-Paul Wagenaar, Robert Waltereit, Liesbeth De Waele, Anna C Jansen

**Affiliations:** 1 Centre for Autism Research in Africa Division of Child and Adolescent Psychiatry University of Cape Town Cape Town South Africa; 2 Department of Philosophy Faculty of Art University of Antwerp Antwerp Belgium; 3 Mental Health and Wellbeing Research Group Faculty of Medicine and Pharmacy Vrije Universiteit Brussel Brussel Belgium; 4 Association TSC Bourneville France Angers France; 5 School of Psychology University of Birmingham Birmingham United Kingdom; 6 Division of Neurology Department of Pediatrics Cincinnati Children's Hospital Medical Center Cincinnati, OH United States; 7 College of Medicine University of Cincinnati Cincinnati, OH United States; 8 Department of Pediatrics College of Medicine University of Cincinnati Cincinnati, OH United States; 9 Department of Psychology Universidade Federal do Paraná Curitiba, Paraná Brazil; 10 Department of Psychopathology and Mental Health Hospital Pedro de Elizalde Buenos Aires Argentina; 11 Department of Neurology Boston Children's Hospital Boston, MA United States; 12 Japanese Society of Tuberous Sclerosis Complex Tokyo Japan; 13 Associazione Sclerosi Tuberosa APS, ETS Rome Italy; 14 European Tuberous Sclerosis Complex Association Wiesbaden Germany; 15 TSC Canada Mississauga, ON Canada; 16 Department of Pediatrics University of Tennessee Health Science Center Memphis, TN United States; 17 The Boling Center for Developmental Disabilities Le Bonheur Children's Hospital Memphis, TN United States; 18 Rare Disease Research UK London United Kingdom; 19 Patient Research Network Tuberous Sclerosis Association London United Kingdom; 20 Department of Clinical Genetics St George's University Hospital London United Kingdom; 21 TSC Clinic Cincinnati Children's Hospital Medical Center Cincinnati, OH United States; 22 Nuffield Department of Primary Care Health Sciences Medical Sciences Division University of Oxford Oxford United Kingdom; 23 Department of Statistical Sciences University of Cape Town Cape Town South Africa; 24 MuViSU (Centre for Multi-Dimensional Data Visualisation) Department of Statistics and Actuarial Sciences Stellenbosch University Stellenbosch South Africa; 25 Clinical Neurosciences Murdoch Children's Research Institute Melbourne, Victoria Australia; 26 Associação de Esclerose Tuberosa em Portugal Lisbon Portugal; 27 Rosamund Stone Zander Translational Neuroscience Center Boston Children's Hospital Boston, MA United States; 28 Esperity Brussels Belgium; 29 TSC Alliance Silver Springs, MD United States; 30 Society of Parents of Children with Autistic Disorders (SOPAN) Mumbai India; 31 Emma Children’s Hospital Amsterdam University Medical Centers Amsterdam The Netherlands; 32 TAND Expert Centre 's Heeren Loo Noordwijk The Netherlands; 33 TSC Ireland Dublin Ireland; 34 School of Medicine Trinity College Dublin Dublin Ireland; 35 Stichting Tubereuze Sclerosis Nederland (STSN) Volendam The Netherlands; 36 LWL-University Hospital Hamm for Child and Adolescent Psychiatry Ruhr University Bochum Hamm Germany; 37 Department of Psychiatry, Psychotherapy and Preventive Medicine LWL-University Hospital Ruhr University Bochum Bochum Germany; 38 Child and Adolescent Psychiatry LWL-Klinikum Marsberg Marsberg Germany; 39 Department of Paediatric Neurology University Hospitals Leuven Leuven Belgium; 40 Department of Development and Regeneration KU Leuven Leuven Belgium; 41 Department of Pediatrics Koningin Mathilde Moeder- en Kindcentrum Antwerp University Hospital Antwerp Belgium; 42 Department of Translational Neurosciences University of Antwerp Antwerp Belgium

**Keywords:** caregiver well-being, TAND severity trajectories, TAND Toolkit app, TAND, TANDem-2 project, TSC, TSC-associated neuropsychiatric disorders, tuberous sclerosis complex

## Abstract

**Background:**

Tuberous sclerosis complex (TSC) is a rare genetic disorder caused by pathogenic variants in the *TSC1* or *TSC2* genes. Apart from multisystem physical manifestations, most individuals with TSC experience TSC-associated neuropsychiatric disorders (TAND). Little is known about how TAND severity changes over time and what factors may predict these changes. Preliminary data suggest the presence of differential TAND severity trajectories. Caregiver well-being may act as a mediator of TAND severity, and a well-being intervention designed for caregivers of children with developmental disabilities may improve caregiver well-being.

**Objective:**

The study aims are to (1) examine longitudinal trajectories of TAND severity in a large sample of individuals with TSC and to examine potential predictors of differential trajectories, (2) evaluate the association between caregiver well-being characteristics, TAND severity, and severity trajectories, and (3) adapt and evaluate the feasibility, acceptability, and potential efficacy of a brief, online group–based well-being intervention for family caregivers.

**Methods:**

For the first 2 aims, 500 individuals with TSC or their caregivers will be recruited in an accelerated longitudinal design to document TAND severity at 5 time points over 12 months via a web-based app. At each time point, participants will complete demographic, TSC characteristics, intervention, and well-being questionnaires. Data will be analyzed using latent class mixed and multinomial regression modeling (aim 1) and structural equation and mediation modeling (aim 2). Participatory methods will be used to adapt an existing caregiver well-being intervention for the TSC community (aim 3). Thirty caregivers will be invited to participate in the adapted group-based online well-being intervention.

**Results:**

This study was funded from July 2024 (HT94252410790 and HT94252410791), and ethics approvals were obtained from the University of Cape Town (July 2024), Vrije Universiteit Brussel (November 2024), and the Department of Defense Office of Human Research Oversight (December 2024). The TAND Toolkit app was adapted for longitudinal data collection (aims 1 and 2). Recruitment started in December 2025 and will continue until 500 participants are enrolled (anticipated December 2026). Primary outputs are expected by July 2028. For aim 3, experiential and adaptation workshops were completed in June 2025, the pilot intervention was delivered in November 2025, and data collection will continue till May 2026. Outputs are expected by December 2026.

**Conclusions:**

Identification of differential longitudinal TAND trajectories and their correlates will stimulate research in TSC and generate evidence for the self-report quantified TAND checklist as a clinical outcome measure. Understanding the association between caregiver well-being and TAND severity will provide support for targeted well-being interventions. A successful pilot trial will provide preliminary data for larger-scale clinical trials, with the potential to support caregivers and improve TAND outcomes. Together, the findings from the study will help close the gap in interventions for TAND.

**Trial Registration:**

ClinicalTrials.gov NCT06879665; https://clinicaltrials.gov/study/NCT06879665

**International Registered Report Identifier (IRRID):**

DERR1-10.2196/91726

## Introduction

### Overview

Tuberous sclerosis complex (TSC) is a rare genetic disorder caused by pathogenic variants in the *TSC1* or *TSC2* genes, leading to overactivation of the mammalian target of rapamycin intracellular signaling pathway [1,2]. With a birth incidence around 1:6000 [3,4], TSC is associated with multisystem manifestations including tumor formation in the skin, heart, kidney, and brain, high rates of epilepsy, and a wide range of TSC-associated neuropsychiatric disorders (TAND) [1,5,6]. The term TAND refers to the broad range of behavioral, psychiatric, intellectual, academic, neuropsychological, and psychosocial difficulties that can occur in people with TSC [6]. While not all individuals with TSC experience every aspect of TAND, the vast majority experience some TAND manifestations across the lifespan and require targeted identification and support [6,7]. TAND has been identified by TSC families as one of the greatest burdens of the disease and highlighted as a top priority for research [6,8,9]. In a Belgian participatory project to identify research priorities, improving access to treatment for TAND was identified as the top priority [10].

Although more than 90% of individuals with TSC have a lifetime history of TAND manifestations [11], very few receive access to diagnostic or interventional services or support. To reduce this gap, the 2012 Neuropsychiatry Panel coined the term “TAND” (1) as an umbrella term for the wide range of bio-psycho-social challenges seen in TSC and (2) to introduce a shared language for clinical practice and research. TAND includes 6 different “levels” of investigation: behavioral, psychiatric, intellectual, academic, neuropsychological, and psychosocial [12]. At the request of TSC stakeholders, a TAND checklist (TAND-Lifetime checklist [TAND-L])—a tool to support clinicians with a systematic screening for TAND across these levels—was developed, pilot validated, and disseminated in the TSC community [13,14].

One of the characteristics of TAND is its highly heterogeneous nature. Some individuals with TSC have many and severe TAND manifestations, while others may have very few [6,11,12,15]. In an attempt to reduce this “overwhelming uniqueness” of TAND (the fact that everyone with TSC seemed to have their own unique TAND profile), data collected by the TAND-L were analyzed using cluster analytic methods in search of natural “TAND clusters” [16-18]. Ward’s cluster analysis, 1000-fold bootstrapping, and exploratory factor analysis were used to identify 7 natural TAND clusters—autism-like, eat/sleep, dysregulated behavior, mood/anxiety, neuropsychological, overactive/impulsive, and scholastic clusters [16-18]. In a next-step project, Alperin and colleagues [11] independently confirmed these natural cluster profiles and extended the field to examine “clusters-of-clusters.” In a sample of >600 individuals with TSC, they described a group of individuals who had difficulties in all 7 natural TAND clusters (“high symptom burden”), a group with no cluster difficulties (“low symptom burden”), and 5 groupings in between these extremes. These studies represented important steps toward reducing the apparent heterogeneity of TAND with implications for clinical practice and research.

Following the development of the TAND-L and identification of natural TAND clusters, participatory research with the TSC community identified the need for a TAND checklist that can be self-reported by families and could quantify TAND severity. Such a checklist could be used in clinical practice and in research to monitor change in TAND over time and potential outcomes from interventions. Family stakeholders asked for the checklist to be built into a smartphone app and for a toolkit of useful tips and information to use at home as “next steps” for action [16,19]. This was the basis for the TANDem-1 project [19].

### From TANDem-1 to TANDem-2: Closing the Gap to TAND Interventions Through Participatory Research

In TANDem-1, the TAND consortium (an international group of TSC clinical research and lived experts with a focus on TAND research [19]) converted the TAND-L to a self-report quantified checklist (self-report quantified TAND checklist [TAND-SQ]) that individuals with TSC or their caregivers could use to document and quantify TAND difficulties themselves [20]. The TAND-SQ was feasible and acceptable to the TSC community, particularly families, who are the primary intended respondents for the TAND-SQ [20]. In the TAND-SQ, a 0-10 severity rating was added to all TAND cluster items, allowing the quantification of TAND difficulties experienced over the past month. Table 1 summarizes the range of TAND severity scores that can be derived using the severity ratings from the TAND-SQ, as proposed by the TAND consortium [21]: an individual item severity score, a mean cluster severity score (CSS_mean_), a maximum cluster severity score, a mean total TAND severity score (TTSS_mean_), and a maximum total TAND severity score (TTSS_max_). The CSS_mean_ has been found to be internally consistent and significantly related to relevant clinical diagnoses and standardized behavioral measures. The TTSS_mean_ has also been found to be a valid indicator of overall functioning and behavior [21].

**Table 1:** Tuberous sclerosis complex–associated neuropsychiatric disorders (TAND) severity scores that can be derived from the self-report quantified TAND checklist.

Score name	Description	Maximum score	Proposed purpose
Individual item severity score (IISS)	TAND severity score of an individual item (eg, sleep)	10	Most useful to track an individual TAND manifestation of interest
Mean cluster severity score (CSS_mean_)	The arithmetic mean score of a specific TAND cluster (eg, autism-like cluster)	10	Most useful to compare one mean cluster score to another with the same maximum score of 10
Maximum cluster severity score (CSS_max_)	The arithmetic sum of all severity scores within a specific TAND cluster (eg, all 4 items in the scholastic cluster)	Maximum scores vary by cluster, given the varying number of items per cluster	Most useful to track the total severity of a specific cluster
Mean total TAND severity score (TTSS_mean_)	The arithmetic mean of all severity scores across all TAND clusters	10	Most useful to track or examine overall TAND severity
Maximum total TAND severity score (TTSS_max_)	The arithmetic sum of all severity scores across all TAND clusters	330^a^	Most useful to track or examine overall TAND severity

^a^The maximum will be 290 if question 6 (difficulties in school) is not completed for those of preschool age.

As part of the TANDem-1 study [19], the TAND-SQ was built into a smartphone app for ease of access, along with a TAND toolkit containing recommendations [22] on what additional help families might seek for TAND, and practical evidence-informed strategies they might implement themselves at home. On conclusion of the TANDem-1 project, the consortium acknowledged a number of scientific knowledge gaps that could be pursued as next steps. These included the need to consider how TAND severity may change over time, how caregiver well-being may be associated with TAND severity and severity trajectories, and the need to generate an evidence base to support the well-being of family caregivers in the TSC community.

### Longitudinal Trajectories of TAND Severity and Their Potential Predictors

At present, there are almost no data about the natural trajectory of the severity of TAND manifestations over time, and this has been identified as an important area for TAND research [15]. There is a small body of literature related to autism, social-communication development, and behavior in TSC, where studies have investigated the 0-36 months age group in relation to biomarkers for autism [23-25]. In earlier studies, the Tuberous Sclerosis Registry to Increase Disease Awareness (TOSCA) consortium [26] examined TAND manifestations stratified by age (children vs adults), and across 7 age bands [12]. Many significant differences were observed with higher rates of TAND manifestations in children vs adults [12]. However, on further examination of age-based profiles in relation to intellectual ability (IA)—known to be a strong correlate of many neuropsychiatric features—results were significantly different [27]. Once controlled for IA, children had higher rates of overactivity, but most behavioral difficulties were higher in adults. At the psychiatric level, rates of attention-deficit/hyperactivity disorder (ADHD) were higher in children, but anxiety and depressive disorders were higher in adults. Controlling for IA, males had higher rates of overactivity and impulsivity as well as ADHD and autism. Importantly, no genotype-TAND correlations were seen on any behavioral, psychiatric, or academic manifestations after controlling for IA [27]. These findings were a stark reminder of the limitations of cross-sectional correlations of TAND and the need for a standardized and quantified tool that can measure change in severity over time.

Apart from the clear research evidence of high levels of heterogeneity in TAND profiles between individuals with TSC, there is also clinical evidence of significant interindividual and intraindividual variability in those outcome patterns over time. However, the majority of TSC studies to date (including clinical trials in TSC) have “averaged out” those health outcomes across study samples or in prespecified stratified subgroups of individuals (eg, Examining Everolimus in a Study of Tuberous Sclerosis Complex-1, 2, and 3) [28-31]. Therefore, describing populations of individuals with TSC using averaged estimates leads to the risk of oversimplifying the complex interindividual and intraindividual variability of real-life TSC [32]. For example, in any intervention, an assumption is typically made that the outcome variable of interest is relatively stable over time and that the statistical change observed will be attributable to the “active ingredient” of the intervention. However, it is likely that there will be naturalistic changes in many outcomes over time. These changes may be associated with differential trajectories of groups of individuals. In the case of TAND, it is very important to consider changes in severity over time, given that TAND severity is highly variable. While it may present a stable ”mean” trajectory, it may be associated with differential natural trajectories over time. Understanding the natural longitudinal TAND severity trajectory of individuals with TSC and identifying differential trajectories of TAND severity over time are fundamental aspects for TAND intervention research.

**Figure 1:**
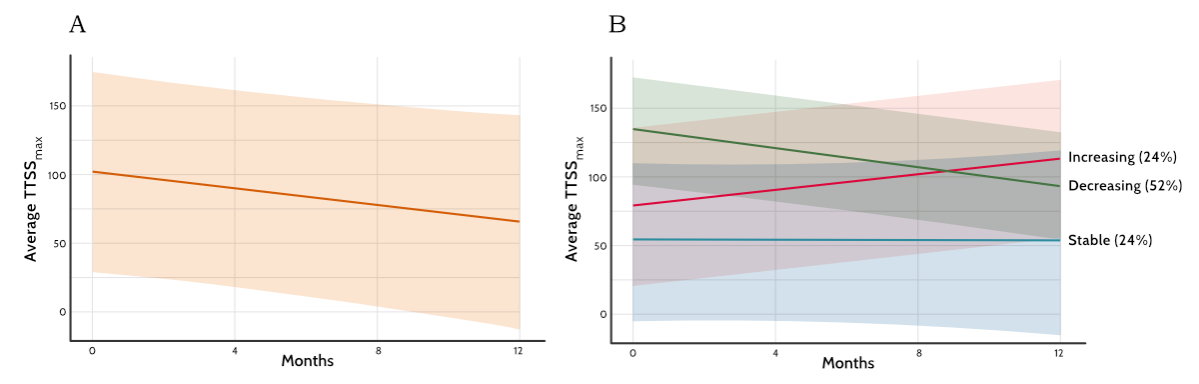
Preliminary data showing differential tuberous sclerosis complex–associated neuropsychiatric disorders (TAND) severity trajectories in 21 participants over a period of 12 months. (A) The overall group maximum total TAND severity score (TTSS_max_) trajectories over the 12-month period with an average decline of 1% per month in TTSS_max_ over time. (B) Three different TAND severity trajectories with decreasing TTSS_max_ in 52% (11/21) of participants, increasing TTSS_max_ in 24% (5/21) of participants, and stable TTSS_max_ in 24% (5/21) of participants.

To begin exploring this question, we performed preliminary analyses of TAND severity over time using data from the TAND-SQ validation study [21], which included 21 repeat completions of the TAND-SQ. The checklists were completed with a mean age of 9.7 (SD 3.63; range 4-15) months apart. A total of 18 checklists were completed by caregivers of individuals with TSC reporting on their child (aged <18 years, n=12), or adult (aged >18 years, n=6) dependents, and 3 were individuals with TSC reporting for themselves. For analysis of change in TTSS_max_, we used the time of the second observation as integer months since the first observation (Table 1). Linear mixed-effects model fit by restricted maximum likelihood, including subject-specific random effects, showed that the mean TTSS_max_ was 100.5 (SD 64.2) at baseline, and that it changed by –1.05 units per month, equating to a 1% decline per month (10 units over 10 months or 12 units over 12 months). These findings therefore showed a “group-based” finding of gradual decline in the TTSS_max_ over time (Figure 1A), suggesting improvement in TAND severity over time. To explore the possibility of latent classes of change trajectories, we set a 10-unit change as a clinical marker of meaningful change (given that 10 units is the total of 1 severity quantification range per item). We grouped the 21 sets of observations into 3 groups based on whether trends between the 2 time points showed a decrease of >10 units, stayed within 10 units, or showed an increase of >10 units. We then regressed the TTSS_max_ per month, allowing for interaction with the 3 classes (stable, increasing, and decreasing). Results (Figure 1B) showed that 5 (24%) participants remained within 10 units from a relatively low baseline (T_0_ mean TTSS_max_ 52.6, SD 46.6), 5 (24%) participants had scores that increased by >10 units from a higher baseline (T_0_ mean TTSS_max_ 77.4, SD 70.0), and 11 (52%) participants had scores that reduced by >10 units from a high baseline (T_0_ mean TTSS_max_ 133.1, SD 63.2). These preliminary findings support the hypothesis of potential differential trajectories in TAND severity over time.

A range of individual factors have been identified that relate to TAND severity, including age, IA, genotype, and seizure characteristics, among others. While the impact of seizures and intellectual disability on TAND outcomes is widely accepted, neither seizures nor any of the other previously examined individual factors have been shown to be necessary or sufficient to cause any specific TAND manifestations or TAND clusters [5,25,33-35]. It is possible that the identification of differential TAND severity trajectories may help to clarify the role of these previously identified predictors; specifically, predictors may play different roles in different trajectory pathways. This is an important research question in TSC with direct relevance to clinical disease tracking and to inform pharmacological and nonpharmacological intervention studies of TAND.

### Caregiver Well-Being and TAND Severity

There is a growing evidence base for the psychosocial burden of TSC and TAND on individuals with TSC and their caregivers [9,36-39]. For this reason, the recent evidence-informed consensus recommendations for the identification and treatment of TAND [22] included a new “wraparound psychosocial cluster” in addition to the 7 natural TAND clusters previously identified [16-18]. However, the association between the psychosocial and other TAND clusters has received little attention in research to date. For this reason, the TAND-SQ included a question that contains 7 items on psychosocial difficulties experienced by the individual with TSC (question 8.1) and by their caregiver (question 8.2). Items enquire about low self-esteem, stress in the family, stress in relationships with siblings, parent-child relationship difficulties, parent-to-parent/partner relationship difficulties, stress leading to difficulty for the family to connect with others in their community, and career progress [20].

As part of the development of the TAND-SQ, data were collected through the TSC Alliance electronic self-report portal. This portal was added as a substudy to the existing TSC Alliance Natural History Database which allowed families or individuals to report on their own health outcomes [40,41]. TAND-SQ responses from 51 caregivers showed very high rates of psychosocial burden (Table 2). Notably, nearly 80% (40/51) of caregivers reported very high levels of stress in the family, and the majority reported very high parent-to-parent (26/51) and parent-child stress (28/51), as well as very high levels of stress making it difficult for the family to connect with others in the community (26/51).

**Table 2:** Proportion of participants endorsing items related to caregiver psychosocial burden (question 8.2) on the self-report quantified tuberous sclerosis complex–associated neuropsychiatric disorders checklist (TAND-SQ; n=51).

TAND-SQ question 8.2 items	Values, n (%)
Low self-esteem	20 (39)
Very high family stress	40 (78)
Very high sibling stress	15 (29)
Very high parent-child stress	28 (55)
Very high parent-parent stress	26 (51)
Difficulty connecting in the community	26 (51)
Difficulty progressing in career	23 (45)

**Figure 2:**
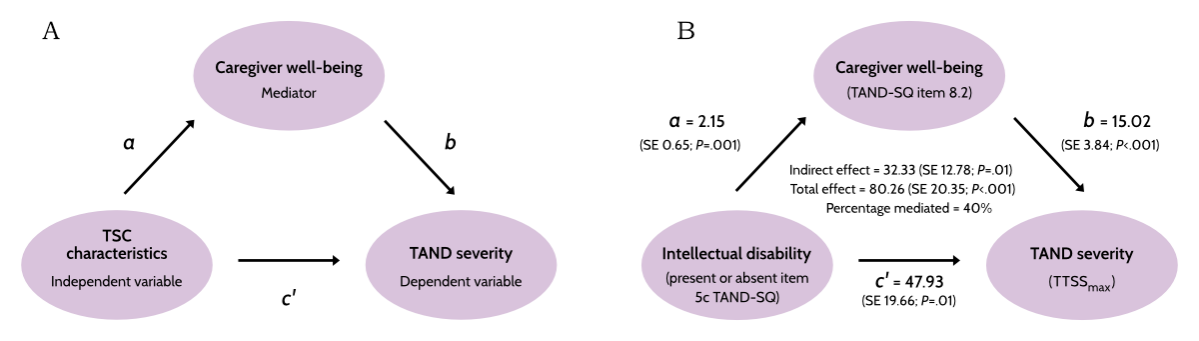
Proposed tuberous sclerosis complex (TSC)–associated neuropsychiatric disorders (TAND) caregiver mediation model. (A) The proposed TAND caregiver mediation model. (B) Summary of the structural equation modeling results (n=51), where caregiver well-being was shown to mediate 40% of the relationship between intellectual disability and TAND severity. TAND-SQ: self-report quantified TAND checklist; TTSS_max_: maximum total TAND severity score.

In the TSC community, caregiver burden has, to date, been conceptualized as a *consequence* of the severity of their family member’s physical TSC manifestations and TAND profile. Interventions have therefore been recommended to support improvement in TAND directly, both to reduce the severity of TAND and therefore caregiver burden. To a lesser extent, interventions to support caregivers and improve their coping skills have been recommended. However, there have been no investigations of the impact of caregiver well-being factors on the TAND severity of their family member with TSC, or to consider whether caregiver factors may mediate TAND severity outcomes (Figure 2A). A mediator is a variable that explains the impact of an independent variable (eg, intellectual disability) on a dependent variable (in this case, TAND severity). The implication of understanding caregiver well-being as a mediating factor is that interventions to support family caregivers’ well-being may have an impact not only on the caregiver directly but also indirectly on the TAND severity of their family members with TSC, thus making caregiver well-being factors “active ingredients” in the pathway toward closing the gap to treatment for TAND.

In conditions outside rare genetic disorders such as TSC, findings are emerging about the fundamental role of caregivers and caregiver factors as mediators of the severity of their family members’ neuropsychiatric profile [42-44]. There have been no investigations of the impact of caregiver well-being factors on the TAND severity of their family members with TSC to consider whether caregiver factors may mediate TAND severity outcomes. A preliminary test of this hypothesis was conducted using data from the 51 participants from the TSC Alliance in the TANDem-1 project mentioned earlier. Structural equation modeling (SEM) was used to determine whether caregiver well-being mediated the relationship between the known TAND severity predictor of intellectual disability and overall TAND severity. Caregiver well-being was measured using the sum of items endorsed in question 8.2 of the TAND-SQ. IA was measured by question 5 of the TAND-SQ and coded as present or absent based on the caregiver’s view (question 5c), and overall TAND severity was quantified by the TTSS_max_. Findings are shown in Figure 2B and indicate that the presence of intellectual disability directly predicted both TAND severity (*c’*=47.93, SE 19.66; *P*=.01) and caregiver well-being (*a*=2.15, SE 0.65; *P*=.001). In addition, caregiver well-being was shown to mediate the impact of intellectual disability on TAND severity (*b*=15.02, SE 3.84; *P*<.001). Findings showed indirect effects of 32.33 (SE 12.78; *P*=.01) and a total effect of 80.26 (SE 20.35; *P*<.001), suggesting that caregiver well-being explained approximately 40% of the variance in TAND severity. This model acknowledges the potential role of individual factors (as independent variables), such as age, sex, genotype, age of seizure onset or control, in the pathway to TAND severity (Figure 2A, path *c’*), and accepts the need for direct and targeted interventions on TAND. However, the novelty of the hypothesis lies in the introduction of a mediation model via caregiver well-being (Figure 2A, path *a* and path *b*) to TAND severity and to different trajectories of severity. These findings provide the first preliminary support for a possible “TAND caregiver mediation model” and the need for further research in this area.

### Strengthening Caregiver Well-Being

There is a global emphasis on policies and programs that support caregiver skills to promote the development of their children and family members with developmental disabilities [45,46]. However, to date, very little attention has been paid to the capacity of caregivers to care [47]. With a limited capacity to care, it is difficult for caregivers to incorporate or implement new knowledge, skills, or techniques, often worsening a caregiver’s sense of competence [48,49].

In the context of these findings and given the highlighted burden of caring for an individual with TSC [9,36,37] (and possible mediation effect of caregiver well-being and TAND severity), we propose a creative solution to improve caregiver capacity to care. Acceptance and commitment therapy (ACT, pronounced as “act”) is an established process-based cognitive behavioral therapy designed to promote psychological flexibility [50]. Psychological flexibility has been defined as “one’s ability to fully attend to the present time, think with openness, and work to advance one’s life in personally meaningful ways” [51]. Tools such as metaphors and experiential exercises are used to help individuals learn how to make healthy contact with difficult thoughts, feelings, memories, and physical sensations that they may have feared or avoided in the past. Participants learn how to make a nonjudgmental space for these painful thoughts, feelings, and memories while developing greater clarity about their personal values and committing to small changes in behavior in line with these values [50]. A growing body of evidence suggests that ACT interventions can result in positive changes in psychological flexibility and, in turn, in the psychosocial well-being of caregivers of children with a range of psychological and physical difficulties, including autism, chronic pain, and significant health needs [52-57]. The findings of these studies and reviews support ACT as a transdiagnostic intervention and suggest that even very brief interventions can have psychosocial benefits for caregivers [58,59]. In TSC, no studies to date have examined the potential feasibility of ACT to support caregiver well-being.

The World Health Organization (WHO) drafted a prepublication stand-alone Caregiver Well-being module as part of the WHO Caregiver Skills Training Program for caregivers of children with developmental delays, disabilities, or disorders [60,61]. The module consists of three 2-hour sessions where 2 trained facilitators meet once a week with a group of about 10 caregivers. Facilitators use a manualized facilitator guide to deliver the intervention. In partnership with the WHO, researchers in South Africa used a participatory approach to perform the first adaptation and evaluation of the Caregiver Well-being module with South African families with children who had a range of developmental disabilities. The work resulted in the novel “Well-Beans for Caregivers” intervention, which was delivered in a low-resourced rural community in one of the South African provinces to 10 caregivers, including 1 with a child who had TSC [62]. A specialist facilitator led the intervention, cofacilitated by a nonspecialist (the mother of a child with autism and employee of the national autism nonprofit organization in South Africa). Caregivers had a mean age of 41.27 (SD 11.07) years, almost all of them were single parents, and had no tertiary education. Their children were an average of 8 (SD 2.36; range 4-11) years of age, with diagnoses including autism, intellectual disability, ADHD, epilepsy, and TSC. Almost half had developmental delays in motor milestones, recapitulating a profile very similar to that seen in TSC. Feasibility was evaluated with attendance tracking, completion rates of digital data collection forms, feasibility evaluation of group sessions, and competence of the specialist facilitator (rated by nonspecialist facilitators and observers). Acceptability was evaluated through acceptability ratings of group sessions by caregivers, facilitators, and observers. Potential efficacy was evaluated using 6 standardized and validated measurement tools, including the Acceptance and Action Questionnaire II, a measure of psychological flexibility [63], the Patient Health Questionnaire-9 (PHQ-9), a measure of parental depression [64], the Generalized Anxiety Disorder 7-item scale (GAD-7) [65], the Multidimensional Scale of Perceived Social Support (MSPSS), a measure of caregiver perception of community support [66], the Family Impact of Childhood Disability Scale (FICDS), a measure of positive and negative appraisal of disability [67], and the Brief Measure of Parental Well-being [68].

Results showed very high attendance rates for each of the 3 sessions (session 1: 9/10, 90%; session 2: 10/10, 100%; session 3: 9/10, 90%), and form completion was 100%. The feasibility of group sessions was rated as very high for facilitator competency (mean 92.67, SD 6.43) and training of others (mean 93.33, SD 4.5). Acceptability of the groups was rated as high by the caregivers, facilitators, and observers. Despite the small sample size, pre-post efficacy measures showed medium to large effect sizes (r, where r=Z/N is the nonparametric effect size) on 7 of the 10 measures, and large effect sizes (r>0.5) on 5 measures, including the PHQ-9 (r=0.52), GAD-7 (r=0.56), MSPSS Total (r=0.58), and FICDS (positive impact, r=0.64). These preliminary data showed strong early-phase evidence to proceed to the next step in the clinical trials pipeline [62].

In summary, there is a clear need in the TSC field to build on the findings of the TANDem-1 project and current TAND literature by exploring longitudinal trajectories of TAND severity and what personal factors and TSC characteristics may predict these trajectories. This is important to inform clinical care and make prognostications about TAND severity. Research is also needed to better understand caregiver well-being in TSC and how it may act as a mediator of TAND severity and TAND severity trajectories. Elucidating these relationships is important for identifying targets of treatment for TAND severity. Finally, it is of utmost importance to learn more about how to support caregivers in their chronic caregiving journey. These needs are the basis for the aims of the TANDem-2 project.

The TANDem-2 project will be carried out by the TAND consortium, originally formed in the TANDem-1 project and expanded for TANDem-2 to consist of 33 adult members (>18 years of age) from 16 different countries around the world, with 11 members being family representatives (individuals with TSC or family members of individuals with TSC). Other consortium members include TSC professional stakeholders, technology developers, global TSC stakeholders, and established and emerging TAND researchers. Here we present the aims, objectives, measures, data collection procedures, data analysis methods, data protection, data management, and ethical considerations for the TANDem-2 project.

### Aims, Objectives, and Hypotheses of TANDem-2

#### Overview

The study aims and hypotheses are outlined below. Specific objectives linked to the aims are shown in Textbox 1.

**Aims and objectives of the TANDem-2 project.**

**Aim 1: Examine longitudinal trajectories of tuberous sclerosis complex (TSC)–associated neuropsychiatric disorders (TAND) severity and predictors of differential trajectories**

- 1.1: Adapt the TAND Toolkit app for longitudinal data collection.
- 1.2: Collect the self-report quantified TAND checklist and other relevant questionnaires longitudinally from 500 participants at 5 time points over 12 months.
- 1.3: Use data collected in 1.2 to identify longitudinal trajectories of TAND severity and potential predictors of differential trajectories.

**Aim 2: Examine caregiver well-being as a possible mediator of TAND severity and severity trajectories**

- 2.1: Using data collected in aim 1, examine the relationship between caregiver well-being, TAND severity, and its trajectories.

**Aim 3: Adapt and evaluate the feasibility, acceptability, and potential efficacy of a brief caregiver well-being intervention**

- 3.1: Use participatory methods (experiential and adaptation workshops) to adapt the “Well-Beans for Caregivers” intervention materials specifically for family caregivers of individuals with TSC of any age, as well as the methods for online delivery.
- 3.2: Evaluate the feasibility, acceptability, and perform limited efficacy testing of the adapted “Well-Beans for Caregivers” intervention in a small pilot trial.

#### Aim 1: Examine the Longitudinal Trajectories of TAND Severity and Their Predictors

We hypothesize that longitudinal analysis of TAND severity data will identify at least 3 differential trajectories (“stable,” “worsening,” “improving”), and that trajectory membership will have differential predictors. To test this hypothesis, we will use an accelerated longitudinal design to collect longitudinal data from 500 families across 5 time points with 3-month intervals over a 12-month period.

#### Aim 2: Examine Caregiver Well-Being Factors as Mediators of TAND Severity and TAND Severity Trajectories

We hypothesize that cross-sectional mediation modeling will identify specific caregiver well-being factors that mediate or moderate TAND severity of TSC family members, and that sequential causal mediation analysis of those factors will identify predictors of longitudinal TAND severity outcomes over time. These specific caregiver well-being factors could include caregiver psychosocial burden, caregiving burden, levels of resilience, depression or anxiety, or overall well-being. To test this hypothesis, the caregiver dataset generated for aim 1 will be used. First, SEM will be used for mediation modeling of baseline data (T_0_). Next, trajectory membership and their predictors identified in aim 1 will be combined with the caregiver mediator factor confirmed through SEM to perform sequential causal mediation analysis.

#### Aim 3: Adapt and Perform a Pilot Feasibility and Potential Efficacy-Testing of an Innovative Brief Caregiver Well-Being Intervention

We hypothesize that an innovative, brief caregiver well-being intervention developed for caregivers of children with developmental disabilities can be adapted as a caregiver well-being intervention in TSC, that it can be delivered feasibly in an online modality, and that we will observe signals of change in key caregiver well-being factors identified in the longitudinal trajectory (aim 1) and mediation analyses (aim 2). To test these hypotheses, we will use participatory methods with the TAND consortium to adapt the 3-session “Well-Beans for Caregivers” program for the TSC caregiver community. After adaptation, we will deliver the 3-session intervention online to 3 groups of 10 caregivers each using a quasi-experimental pre-post follow-up design including measures of the key caregiver well-being factors examined in the mediation modeling and additional measures specific to the intervention. As mandated by our funding agency, only a pilot trial will be conducted.

**Figure 3:**
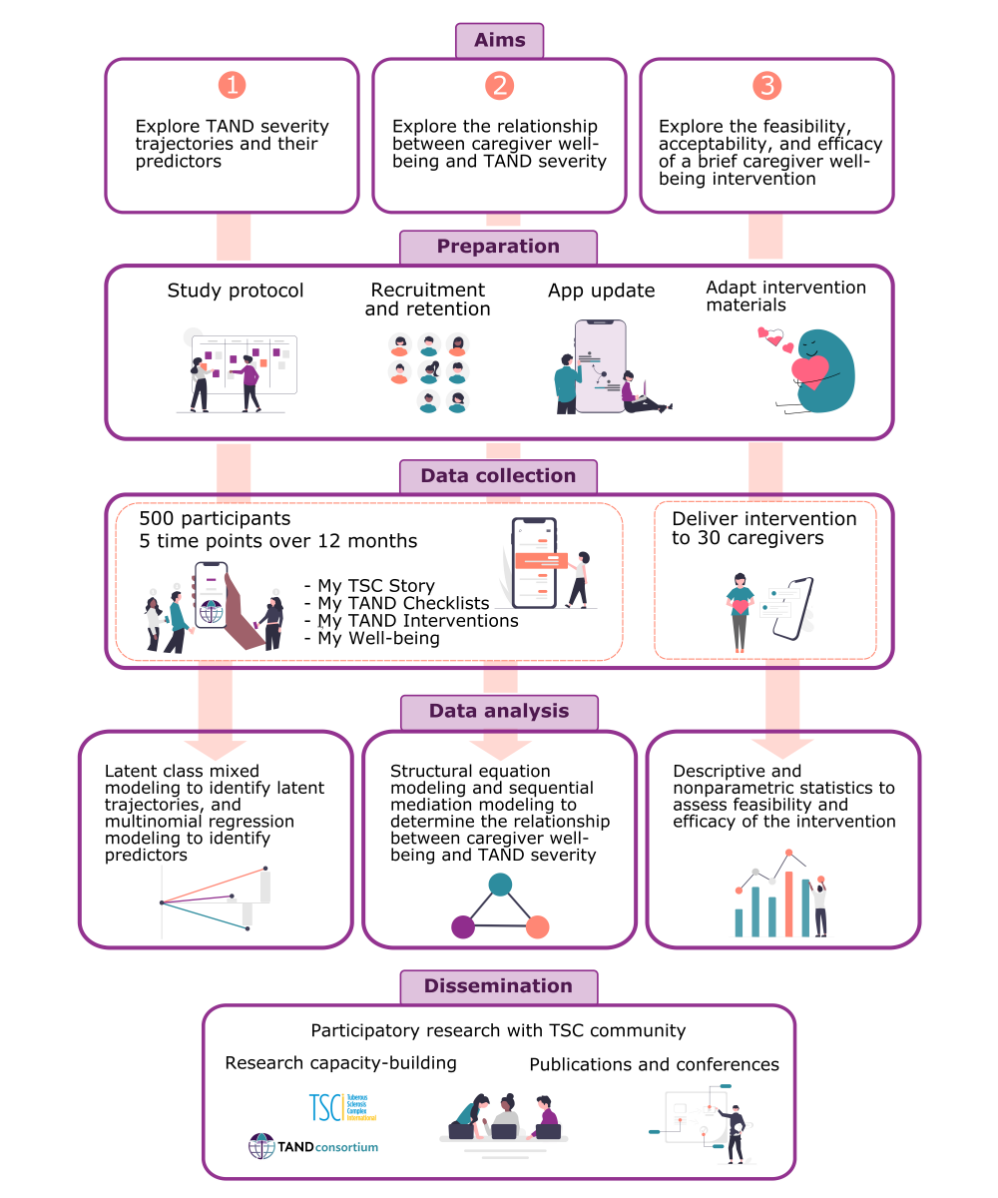
A visual summary of the TANDem-2 project. TAND: tuberous sclerosis complex–associated neuropsychiatric disorders; TSC: tuberous sclerosis complex.

A visual summary of the TANDem-2 project is provided in Figure 3.

## Methods

### Study Participants Aims 1 and 2

#### Inclusion Criteria

A total of 500 adult participants (>18 years of age) will be recruited. Inclusion criteria are (1) adult individuals with TSC of any age, sex, genotype, and an intellectual level that will allow them to participate in the study; (2) adult caregivers (>18 years of age) of individuals with TSC (of any age, sex, genotype, and intellectual level); (3) willingness to provide informed consent; (4) willingness and ability to complete data collection at 5 time points over a period of 12 months using the TAND Toolkit app; (5) access to a smartphone or other digital device (eg, tablet, laptop, or desktop computer) and mobile data or internet access to complete data collection using the app; (6) sufficient knowledge of technology to be able to access the web-based smartphone app on their digital devices; and (7) sufficient understanding of English to be able to understand the study information and consent documents, and to complete the English questionnaires included in the study.

#### Recruitment

Study participants will be recruited through TSC International (TSCi), the umbrella organization for all international TSC nonprofit organizations. TSCi represents a total of 38 individual organizations across the globe, including the TSC Alliance, based in the United States. The TSC Alliance has a global reach for research recruitment, including the Natural History Database, which includes over 2700 participants at the time of writing [40,41]. Family representatives within the TAND consortium are active advocates within their country-specific patient organizations, who will assist with recruitment efforts. Within the TAND consortium, we will cocreate a study flyer with a description of the study and an invitation to participate. The flyer and invitation to participate will be sent electronically by the TSC Alliance and other TSCi partner organizations to TSC families in their networks via their websites, mailing lists, and other organizational communication strategies. Word-of-mouth and snowball sampling may also play a part once recruitment begins.

#### Enrollment

Individuals who wish to participate in aims 1 and 2 of the TANDem-2 research project will be required to register online and use the TAND Toolkit app for data collection. Informed consent will be captured electronically. To optimize potential participant understanding of study participation requirements, and in line with current recommendations regarding electronic consent processes [69-72], we have incorporated various elements in the recruitment and enrollment process. Interested participants will be able to click on a link or scan the QR code on the flyer to access further information about the study on a dedicated web page on the TAND consortium website [73]. This study’s web page will contain detailed information about the study in various formats: a short video about the TANDem-2 project, a visual summary of the steps involved in data collection, a PDF document of the approved study information, and consent form, as well as the privacy policy and terms of use for the TAND Toolkit app, a set of accessible frequently asked questions, and an email address where any study related questions can be directed to the research team. If interested individuals would like to join this part of the study, they can click on the button at the bottom of the project information web page to proceed to the TAND Toolkit app registration web page. The first step will be to read and complete the digital study information and consent form, as well as the app privacy policy and terms of use. The participant will then proceed to complete the registration process and create an app user account. The participant (app user) will then receive an email to verify their account registration, and thereafter will receive access to the TAND Toolkit app. These registration steps are in line with current recommendations to enhance data integrity while using an exclusively digital data collection method [74-79].

#### Retention

In light of the risk of attrition in longitudinal research, retention will be facilitated by a range of retention strategies shown to be effective in large-scale longitudinal studies [80]. These will include inviting more than the target number of participants to enroll (up to 600), incentivization through invitations to study webinars, and study updates and activities linked to TSC Alliance and TSCi events. Participants will not be compensated for participation, another key feature for enhancing data integrity in digital research [78]. Further technical retention strategies built into the TAND Toolkit app are described in the measures and data collection methods for aims 1 and 2 under the “Measures and Data Collection” section.

### Study Participants (Aim 3)

#### Inclusion Criteria

For objective 3.1 (adaptation), all members of the TAND consortium, prioritizing those with lived expertise in TSC, will be invited to participate in the adaptation of the “Well-Beans for Caregivers” intervention by participating in 3 experiential sessions of the intervention and an adaptation workshop. All consortium members will be required to sign informed consent prior to participation. Inclusion criteria for objective 3.2 (pilot intervention), potential study participants are (1) adult caregivers (>18 years of age) of a family member with TSC (with any age, sex, genotype, and intellectual level); (2) willingness to provide electronic informed consent; (3) willingness and ability to participate in 3 online intervention sessions (each session will last 2 hours, 1 session per week over 3 weeks); (4) willingness and ability to complete pre-, post-, 3-, and 6-month follow-up data collection; (5) need to have access to a digital device (eg, smartphone, tablet, laptop, or desktop computer) and mobile data or internet access in order to participate in the online intervention sessions; (6) willingness to join a group intervention session; and (7) sufficient level of English to be able to read information, booklets, and participate in sessions and data collection.

#### Recruitment

For objective 3.2 (pilot intervention), 30 participants will be recruited from 3 international sites (10 participants per site). Participants will be recruited via purposive sampling through invitation by our community partners. The invitation email will explain the requirements for participation and include the approved study information form for review. 

#### Enrollment

Participants who wish to participate will be asked to copy their reply to the research team. They will then be sent an email containing a link to the REDCap (Research Electronic Data Capture; Vanderbilt University) platform to sign consent using the e-Consent feature and complete the preintervention forms. They will also receive information about and links to the scheduled intervention sessions.

#### Retention

Due to the brevity of the 3-session intervention and attendance rates in a previous study with this intervention [62], we do not anticipate significant challenges in retention of study participants for aim 3.

### Intervention Delivery

The adapted intervention will be delivered online by 2 trained facilitators (members of the TAND consortium). Trainee facilitators will attend as observers. Sessions will take place once a week for 3 consecutive weeks, in 2-hour sessions, with a group of 10 caregivers. Sessions will follow the manualized script in the adapted facilitator and caregiver booklets. These include stories, exercises, and group discussions to help convey key ACT-informed psychological principles. The content, discussions, and activities cover the main ACT constructs of (1) getting present, (2) identifying personal values, (3) taking small, committed actions in line with these values, (4) finding ways to take care of yourself, (5) naming and noticing your thoughts and feelings, and (6) acceptance, which collectively make up the construct of psychological flexibility [50,81]. This pilot trial has been registered on ClinicalTrials.gov (NCT06879665).

### Measures and Data Collection

#### Overview

A range of data measures has been developed and selected to define individual, caregiver, and TAND severity characteristics at baseline, during longitudinal data collection, and in the pilot well-being intervention. Measures were selected if they were freely available, comprehensive, and had good psychometric properties.

#### Measures and Data Collection Methods (Aims 1 and 2)

The measures for aims 1 and 2 include demographic information, TSC characteristics, TAND severity, TAND interventions, and information about well-being. All of these measures will be collected using the TAND Toolkit app, adapted for longitudinal data collection and accessibility via various electronic devices (such as smartphones, tablets, or desktop computers). Table 3 summarizes the data measures, app sections, time points at which each measure will be completed, the estimated length of time measures will take to complete, and who the measures capture information about. There will be 5 data collection time points over a 12-month period, with 3-month intervals.

**Table 3:** Data collected using the tuberous sclerosis complex (TSC)–associated neuropsychiatric disorders (TAND) Toolkit app for aims 1 and 2.

Data measures and the type of information collected			TAND Toolkit app section		Data collection time points		Estimated time to complete	Who the measures are completed about
**Demographic information**								
	Age Education level and type Family living situation Employment and income	My TSC Family		1		5 min		Individuals with TSC and the caregiver (if relevant)
**TSC characteristics (My TSC Story)**								
	Age diagnosed First concerns Current physical symptoms in each body system Genetic testing and genotype Seizure characteristics Treatments	My TSC Story		1-5		15 min		Individuals with TSC
**TAND severity (TAND-SQ^a^)**								
	Developmental milestones Behavioral difficulties Psychiatric diagnoses Intellectual ability Scholastic difficulties Neuropsychological difficulties Psychosocial difficulties Priorities, strategies, and strengths	My TAND Checklists		1-5		30 min		Individuals with TSC (and question 8.2 for the caregiver, if relevant)
**TAND interventions**								
	Type of intervention (medications, psychological/behavioral/talking, educational, social, caregiver or family training, or other) Which cluster it was targeting What difference it made	My TAND Interventions		1-5		10 min		Individuals with TSC
**Well-being**								
	Care/caregiving burden Brief Resilience Scale [82] Patient Health Questionnaire-9 [64] Generalized Anxiety Disorder 7-item scale [65] World Health Organization-5 Well-Being Index [83] Overall well-being rating	My Well-being		1-5		10 min		Individuals with TSC (only self-report); caregivers (only self-report)

^a^TAND-SQ: self-report quantified TAND checklist.

To support longitudinal data collection, the app includes built-in features to encourage retention. There will be automated “reminders” for new data collection time points through in-app notifications and emails to participants. Efforts have been made to ensure each data collection time point is not too time-consuming. Data collection for time point 1 will take approximately 60-70 minutes. At time points 2-5 (T_2_-T_5_), the total data collection time should be faster as several items within the My TSC Story, My TAND Checklist, and My TAND Interventions sections will be prepopulated, thus only requiring updates to be completed. We anticipate data collection at time points T_2_-T_5_ to take 30-40 minutes. We will include visual summaries of the data entered by participants, which will allow them to view changes in their TAND severity over time. These are intended to encourage participants to return to the app for data entry at the subsequent time points. The app will generate PDF summaries of information provided by participants that can be downloaded and shared with health care teams. The app contains the TAND toolkit, developed as part of the TANDem-1 project [20], containing a range of evidence-informed suggestions for what additional clinical help to seek and what can be tried at home for symptoms across all TAND clusters. Access to the TAND toolkit is also considered a retention strategy.

#### Measures and Data Collection Methods (Aim 3)

The schedule of enrollment, baseline assessment, intervention, and immediate post-, 3-month post-, and 6-month postintervention assessments is shown in Table 4. In addition to data measures for aims 1 and 2, measures for aim 3 were selected to evaluate the feasibility, acceptability, and potential efficacy of the “Well-Beans for Caregivers” intervention. These additional measures include attendance rate as a measure of feasibility, session feedback forms as measures of feasibility and acceptability, completed by session facilitators, observers, and caregiver participants, and 2 measures of family functioning and quality of life. A measure of psychological flexibility, the hypothesized ACT therapeutic mechanism of change, has also been included, namely the Psy-Flex [81]. All clinical measures will be collected via REDCap before the intervention starts (preintervention), immediately post intervention, and at 3 months and 6 months after the intervention occurred. Session feedback forms will be collected from participants, observers, and facilitators after each session.

**Table 4:** Data collected using REDCap (Research Electronic Data Capture) for aim 3.

Measure				Time point		Time to complete
Caregiver demographic information				Preintervention		10 min
**Feasibility and acceptability**						
	Attendance		After each session		N/A^a^	
	Session feedback forms: caregivers, observers, and facilitators		After each session		5-7 min	
**Potential efficacy of intervention**						
	**Psychological flexibility**					
		Psy-Flex [81]	Pre-, immediately post-, 3-month post-, and 6-month postintervention		3-5 min	
	**Caregiver burden**					
		TAND-SQ^b^ question 8.2 [20]	Pre-, immediately post-, 3-month post-, and 6-month postintervention		3 min	
	**Caregiver well-being**					
		Brief Resilience Scale [82] Patient Health Questionnaire-9 [64] Generalized Anxiety Disorder 7-item scale [65] World Health Organization-5 Well-Being Index [83]	Pre-, immediately post-, 3-month post-, and 6-month postintervention		10 min	
	**Family support and quality of life**					
		Multidimensional Scale of Perceived Social Support [66] Family Impact of Childhood Disability Scale [67]	Pre-, immediately post-, 3-month post-, and 6-month postintervention		15 min	

^a^N/A: Not applicable.

^b^TAND-SQ: self-report quantified tuberous sclerosis complex–associated neuropsychiatric disorders checklist.

### Data Analysis

#### Statistical Analysis Plan (Aim 1)

TAND severity will be quantified using the CSS_mean_ for each cluster and the TTSS_mean_/TTSS_max_ for overall TAND severity. These will be used to identify latent TAND severity trajectories using latent class mixed effect modeling [84]. The number of classes will be chosen based on statistical measures of model fit, the stability of the latent profiles, and the group sizes. Multinomial regression modeling [84] will be used to investigate the association of potential subject-specific covariates with the identified latent classes (predictors of TAND severity trajectories). All analyses will be done using R software (R Foundation for Statistical Computing) [85].

Power calculations are based on the estimation of the time trajectory and comparing different latent trajectories. Based on the data from the preliminary data analyses described earlier of 21 repeat TAND-SQs (42 observations), the power calculations were made in 2 ways. First, based on simulations [86] using a single slope (mean profile), power for sample sizes of n=500 and n=300 was calculated at a slope of -1.05% (power=100%/100%) and at –0.5% (power=97%/81%). Second, simulations were generated for n=500 and n=300 using a 3-trajectory solution with change in trajectories set at +1%, 0%, –1% (power=100%/100%), +0.5%, 0%, –0.5% (power=84%/72%), and 0%, +0.5%, +1% (power=86%/83%). Power calculations allowing for 3 different estimated baseline values of TTSS_max_ (75, 100, and 125) in addition to differing slopes for trajectories, show for n=500, 100% power (with 1% change in trajectories) and 93% power (with 0.5% change in trajectories). A sample size of n=300 using similar power calculations shows 100% power for 1% and 77% power for 0.5% differences in trajectory slopes.

#### Statistical Analysis Plan (Aim 2)

The caregiver dataset will be analyzed as follows: first, SEM will be used for mediation modeling of baseline data (T_1_). Next, trajectory classes and their predictors identified in aim 1 will be combined with mediators confirmed through SEM to analyze the mediated association between predictors and latent trajectory classes using a generalized structural modeling approach. Separate mediation models will be fit for different combinations of individual risk factors, caregiver well-being measures, and TAND severity outcomes. Additionally, we will extend the structural equation model to incorporate multiple individual factors and multiple caregiver well-being measures for specific TAND severity outcomes, including sequential mediation models that use successive measurements of caregiver well-being as the successive mediators [87]. All analyses will be done using R [85,88].

Using our preliminary analyses regarding a possible “caregiver mediation model” with intellectual disability (binary variable) and TTSS_max_ (274 in the TANDem-1 sample; Figure 2), mediated through caregiver stress (maximum of 7 units), assuming effect size for path *a*=2.15 (SE 0.65) and path *b*=15.02 (SE 3.84), 100 observations will have 95% power to detect a mediation effect=40% of the total effect at the 5% level of significance according to Sobel test for mediation [89]. If we decrease the effect sizes for *a* and *b* by 50%, 300 observations will have 86% power to detect a significant mediation effect.

We will control for false discovery rates due to multiple comparisons as a result of potentially multiple models for the multiple outcome-mediator-predictor combinations using the Benjamin Hochberg procedure [90]. The inclusion of relevant covariates in the models will be identified through directed acyclic graphs [91].

#### Statistical Analysis Plan (Aim 3)

Descriptive and nonparametric statistics will be used to summarize the quantitative data on the feasibility, acceptability, and potential effects of the “Well-Beans for Caregivers” intervention. Given that this is a feasibility and limited efficacy testing (“signal seeking”) pilot study, no formal power calculations were performed. Effect size data from this study will be used to perform power calculations for any future clinical trials of the “Well-Beans for Caregivers” intervention.

### Data Protection and Data Management

#### TAND Toolkit App Data

Protection of digital data is a paramount concern in this project. Data collection via the TAND Toolkit app, for aims 1 and 2, is governed by an approved data management plan, data protection impact assessment, data sharing agreement, data processing agreement, nondisclosure agreement with the app developers, privacy policy, terms of use, and cookie policy for the app, and compliance with the General Data Protection Regulation (GDPR) of the European Union, seen as the most stringent standard for data protection. Key data protection features incorporated into the TAND Toolkit app are outlined in Multimedia Appendix 1.

The app fulfills GDPR requirements in that only essential user data are collected, processes are in place to handle app user data requests (such as access, rectification, or erasure of data), and various organizational and technical measures are in place to ensure secure transfer, hosting, access, and protection of the data. The legal basis for capturing and processing data is the user consent that has received independent institutional review board (IRB)/human research ethics committee approval. The app privacy policy informs the app user about the data that will be collected, for what purposes it will be used, and for how long it will be stored, and informs the users of their rights with regard to their data. The app’s terms of use document outline the rules for using the app and the rights and responsibilities of all parties involved, including the data owners and the app users. All anonymized data collected in the app will be freely accessible to the TSC clinical and research community in accordance with procedures outlined in a data sharing agreement. Data retention is also specified in the informed consent form to align with the GDPR regulation, as well as the right to have insight into what data are collected, and the option to export or remove all user data on request.

#### REDCap Data

Data collection for aim 3 will be done using REDCap, a widely used, robust, and secure web application licensed to research entities for study data collection and storage [92,93]. REDCap includes rigorous query management, audit trails, automated reporting, and data exporting to all statistical software packages that will be used in the study.

We acknowledge that breach of privacy or confidentiality is a potential risk in any clinical research, particularly in the pilot intervention trial where 3 groups of 10 participants each will take part and where personal information may inadvertently be shared by other group members. To mitigate against this, participants will be reminded during consent procedures and at the start of each session to guard against the sharing of any private or confidential information outside the group. Participants will also be reminded, as part of informed consent procedures, that there will be a risk of potential personal information sharing by the group. From an administrative and research perspective, anonymized or aggregated data will be made available via controlled access after application and ethics approval, in accordance with FAIR (Findable, Accessible, Interoperable, and Reusable) principles.

### Ethical Considerations

#### Participant Vulnerability and Risks

This study represents minimal risk to participants as outlined by the Office for Human Research Protections [45 CFR 46.110] and the Food and Drug Administration [21 CFR 56.110]. The TANDem-2 project will include participants who may be considered of medium vulnerability. This includes adults with TSC and family caregivers of dependents with TSC of any age. As recruitment of these participants will be managed through patient support organizations for TSC, we feel confident that participants will have access to support throughout the duration of the study. The study is considered minimal risk to participants, given that all data collection is noninvasive and consists of self-reported behavioral and neuropsychiatric information. Minors will not be direct participants, and given that the pilot trial will be a psychosocial intervention with behavioral data as feasibility and impact measures. Participants in the intervention groups may become emotional or distressed during sessions, and this will be managed by the trained facilitators. A referral system will be in place for participants who appear highly stressed during the sessions for further support.

#### Informed Consent

All participants will provide consent before taking part in the study. All members of the TAND consortium will sign a consent to take part in various activities in the project, including for aim 3, objective 3.1 (experiential and adaptation workshops for adapting the “Well-Beans for Caregivers” intervention). For aims 1 and 2, participants will provide informed consent on the TAND Toolkit app following review of the study information form and the app privacy policy and terms of use. All consent statements completed or checked by participants will be date-time–stamped and archived. Caregiver participants in the aim 3 pilot will sign consent via the e-Consent framework in REDCap, where all consent statements will also be date-time–stamped and archived.

#### IRB/Human Research Ethics Approval

The study will follow all principles established by the International Conference on Harmonization of Good Clinical Practice in Research. The initial study protocol (version 1) was approved by the IRB committee at the initiating principal investigator (PI) site (Vrije Universiteit Brussel [VUB]: EC-2024-222/BUN 1432024000179) and the partnering PI site (Human Research Ethics Committee [HREC], University of Cape Town [UCT]: HREC 327/2024). It has also been approved by the funder’s oversight committee, the US Army Medical Research and Development Command Office of Human Research Oversight (OHRO; E05445.1a). This is protocol version 2 following approved amendments at VUB (October 8, 2025) and UCT (November 10, 2025) and OHRO (December 2025). The pilot trial has been registered in the National Institutes of Health (NIH) clinical trials registry with identifier NCT06879665.

#### Additional Safeguards

Any participants who raise concerns about their own mental or physical state and/or that of their family members with TSC will be referred to the relevant contact person at the TSC Alliance and/or TSCi organization. The initiating and partnering PIs, key research team members, and TAND consortium members are experienced clinical researchers and/or members of TSC nonprofit organizations and will have expertise and networking knowledge to support participants if this were to happen. Participants will be reminded as part of informed consent that they are free to withdraw from the research at any stage without the need to provide a reason for their withdrawal.

#### Education in the Protection of Human Research Subjects

All members of the TAND consortium, regardless of their country of base, and all additional research staff will participate in online training on the protection of human research subjects. This will include an online examination of relevant modules to ensure that all researchers have appropriate knowledge of the principles and federal regulations on the protection of human research subjects, as described in the Belmont Report and relevant federal regulations.

## Results

### Study Progress

This grant was awarded in December 2023, and funding started in July 2024 (HT94252410790 and HT94252410791). IRB approval was obtained from the University of Cape Town (July 2024), Vrije Universiteit Brussel (November 2024), and the Department of Defense OHRO (December 2024). The TAND Toolkit app was adapted for longitudinal data collection in aims 1 and 2 throughout 2025. Recruitment flyers were cocreated with the TAND consortium and approved by all IRB committees. “Soft launch” for recruitment took place in December 2025, and the official launch of the TAND Toolkit app took place on Rare Disease Day (February 28, 2026). Recruitment will continue until 500 participants are enrolled (anticipated December 2026). Data collection will continue until all participants have completed 5 rounds of data collection (every 3 months over a 12-month period). We anticipate this to continue until December 2027. For aim 3, the experiential and adaptation workshops (objective 3.1) were completed in June 2025, and the pilot intervention (objective 3.2) was delivered in November 2025 with postintervention data collection until May 2026.

Analysis and write-up of aims 1 and 2 will depend on recruitment targets, with primary outputs anticipated by July 2028. Analysis and write-up of aim 3 findings are anticipated by December 2026.

### Potential Challenges and Solutions

The most likely challenge in this study will be the recruitment and retention of 500 participants for the longitudinal data collection for aims 1 and 2. However, the TAND consortium and research team are well placed in the international TSC community to manage this process, and the 4-year track record of the TAND consortium during the TANDem-1 project is a testament to this. We acknowledge that the power calculations for the trajectory analyses were based on preliminary data with only 21 repeat TAND-SQ observations and that the mediation power analysis came from preliminary data with 51 caregivers. These data therefore provide limited justification for the final statistical analysis plan. However, we have taken a very conservative approach to prepare our power calculations. First, we acknowledge that TSC is a rare disease and that these represent the first-ever data of repeat measures on the TAND-SQ and of caregiver well-being on question 8.2. Second, there was a funder requirement for a prospective statistical analysis plan and power calculations. As outlined in the data analysis section of this protocol, we have therefore used different approaches to do power calculations, including the use of simulations and using very conservative parameters for change measurement. We also acknowledge that the intervention pilot is small, with only 30 participants in a quasi-experimental pre-post design with no control group and no formal power calculation. This approach was selected for 2 reasons. First, the primary aim of the pilot trial was to evaluate feasibility and acceptability with a very limited evaluation of efficacy (“signal seeking” of potential future outcome measures through evaluation of effect sizes of change on theoretically motivated measures) as a secondary aim. Second, there was a funder requirement only to allow small pilot studies to be conducted through this funding mechanism.

### Unexpected Results

Our hypotheses may not be supported by the data collected, but in each case, any alternative findings will be of relevance to the TSC and the scientific field of rare diseases. For example, in aim 1, it is possible that we may not identify 3 latent class trajectories but may find more or fewer trajectories. Such unexpected results will generate novel findings to be explored in relation to baseline/individual factor predictors. In aim 2, it is possible that we may not find statistical support for a strong mediation effect of caregiver well-being factors. Instead, findings may point toward caregiver well-being factors as “moderators” instead of mediators, where caregiver well-being modifies the relationship between individual characteristics and TAND severity rather than explains this relationship. There is growing recognition that a mediator can become a moderator (eg, if specific interventions are introduced to target that particular mediator) [94]. Such findings will provide important new results and potential new hypotheses to explore. Unexpected results in aim 3 may suggest that the adapted “Well-Beans for Caregivers” intervention is not suitable for caregivers of individuals with TSC, or that no signals of potential change are identified in the small-scale pilot study. However, our work in low-resource communities in South Africa with a profile of caregivers of children very similar to those in the TSC community showed high feasibility and remarkably encouraging effect sizes [62], suggesting that similar findings are likely in TSC.

## Discussion

### Overview

The TANDem-2 study aims to build the knowledge base on TAND in TSC by examining longitudinal trajectories in TAND severity and potential predictors of differential trajectories, examining caregiver well-being as a possible mediator of TAND severity and severity trajectories, and adapting and evaluating the feasibility, acceptability, and limited efficacy of a brief caregiver well-being intervention for TSC family caregivers.

### Short-Term Impact

Identification of latent classes of TAND severity trajectories and their predictors will provide scientific data to inform the prognosis of TAND severity and to measure the efficacy of clinical interventions. Awareness of differential TAND severity trajectories will also increase clinical trial readiness with the TAND-SQ as a potential outcome measure. Identification of caregiver well-being factors as mediators of TAND severity in their TSC family members will reconceptualize caregivers as “active ingredients” of TAND severity and provide strong evidence for the importance of “caring for the caregivers” as proposed in the recent international consensus recommendations for the identification and treatment of TAND [22]. This will have an immediate impact on the TSC community, both to explore those caregiver factors in the clinical context and to seek strategies to strengthen them. Based on our earlier work in developmental disabilities [62], caregivers who participate in the intervention will have direct and immediate benefit from participation. Developing a feasible and easily scalable, ultrashort intervention with some signals of efficacy for caregivers will provide strong preliminary evidence for larger-scale nonpharmacological clinical intervention trials with real potential to improve TSC and TAND outcomes, as well as caregiver well-being.

### Long-Term Impact

Identification of differential TAND severity trajectories and their predictors will have a direct long-term impact on scientific research of the underlying mechanisms of action and/or treatment of TAND, where latent class membership can become a novel variable to be explored. Caregivers as mediators will add an additional pathway to TAND severity to be examined and developed. The innovative ultrabrief intervention studied here was designed to be easily scalable. This means that the intervention has the potential for global implementation in a highly scalable and sustainable way following future confirmatory clinical trials. The adapted intervention will be freely available under a Noncommercial Creative Commons License. Together, the findings from the study will clearly provide direct clinical application to the field and close the gap to interventions for TAND with powerful short- and long-term impact.

### Dissemination Plans

Data generated by this proposed study will be shared through collaborations, publications, and presentations at scientific meetings, webinars, and TSC community meetings in the form of oral presentations and posters, and publications in peer-reviewed journals that are widely available. We have included costs for open-access publishing in our budget to ensure the findings are as accessible as possible. Following the NIH Public Access Policy (NOT-OD-08-033), all investigators will submit an electronic version of their final, peer-reviewed work to the National Library of Medicine PubMed Central, to be made publicly available no later than 12 months after the official date of publication.

Research tools and resources in the form of the TAND Toolkit app and adapted “Well-Beans for Caregivers” intervention manuals for TSC will be created during the course of the study. Our aim is to ensure that the TAND Toolkit app will become freely available to the TSC community following the TANDem-2 study. The intervention materials will be made freely available to those interested in being trained to deliver the intervention under a Creative Commons License 4 after completion of the study. This license specifies that the materials are available for use free of charge, but cannot be modified or changed, and acknowledgment must be given to the developers (ie, the TAND consortium). The materials will also be available upon request to qualified academic investigators for noncommercial research. We will work in close partnership with the funders of the project and our project partners to ensure impactful communication and dissemination of findings to the general public and in the TSC community.

## Appendix

Multimedia Appendix 1 Protection features for data collected via the TAND (tuberous sclerosis complex–associated neuropsychiatric disorders) Toolkit app.

## Abbreviations

ACT: acceptance and commitment therapy
ADHD: attention-deficit/hyperactivity disorder
CS: cluster score
CSS_mean_: mean cluster severity score
FAIR: Findable, Accessible, Interoperable, and Reusable
FICDS: Family Impact of Childhood Disability Scale
GAD-7: Generalized Anxiety Disorder 7-item scale
GDPR: General Data Protection Regulation
HREC: Human Research Ethics Committee, University of Cape Town
IA: intellectual ability
IRB: institutional review board
MSPSS: Multidimensional Scale of Perceived Social Support
NIH: National Institutes of Health
OHRO: Office of Human Research Oversight
PHQ-9: Patient Health Questionnaire-9
PI: principal investigator
REDCap: Research Electronic Data Capture
SEM: structural equation modeling
TAND: TSC-associated neuropsychiatric disorders
TAND-L: TAND-Lifetime checklist
TAND-SQ: self-report quantified TAND checklist
TOSCA: Tuberous Sclerosis Registry to Increase Disease Awareness
TSC: tuberous sclerosis complex
TSCi: TSC International
TTSS_max_: maximum total TAND severity score
TTSS_mean_: mean total TAND severity score
UCT: University of Cape Town
VUB: Vrije Universiteit Brussel
WHO: World Health Organization

## Data Availability

On completion of the study and publication of the results, anonymized data will be accessible to the TSC research community through a standardized application process.
