# Supplementary material for: Closing the Gap to Interventions for Tuberous Sclerosis Complex–Associated Neuropsychiatric Disorders (TAND): Protocol for a Longitudinal Study of TAND Severity, Predictors, and Caregiver Well-Being (TANDem-2)
Source: JMIR Res Protoc. 2026 May 5;15:e91726. doi: 10.2196/91726 (PMC13187705; doi:10.2196/91726)
Supplement: Multimedia Appendix 1 [file resprot_v15i1e91726_app1.docx]

**Multimedia Appendix 1**

**de Vries et al (2026). Closing the gap to interventions for TSC-Associated Neuropsychiatric Disorders (TAND): Protocol for a longitudinal study of TAND severity, predictors and caregiver wellbeing (TANDem-2)**

***URL:*** [***https://www.researchprotocols.org/2026/0/e0/***](https://www.researchprotocols.org/2026/0/e0/) ***doi: 10.2196/91726***

### Data protection and data management

#### TAND Toolkit app data

Key data protection features incorporated into the TAND Toolkit App are outlined in **Table 1**.

**Table 1. Protection features for data collected via the TAND Toolkit App**

| **Safety feature** | **Description** |
| --- | --- |
| Registration and App access | - Email addresses will be verified upon account registration - The app is username and password protected - App users have the option to activate two-factor authentication |
| App to server data transfer | - All data exchanges between the app and the data servers are encrypted using Secure Sockets Layer (SSL) / Transport Layer Security (TLS) certificates to ensure secure data transfer |
| Data server | - App data will be hosted on GDPR-compliant servers in the European Union (EU) - All participant-checked consent statements will be date-time-stamped and archived - To protect against unauthorised access, password is hashed, while email is both encrypted and hashed, and all information containing personal identifiable information in encrypted - A web application firewall is deployed to filter and monitor incoming traffic to help prevent malicious activities - Libraries and systems will be regularly updated to address known security vulnerabilities - Server health will be monitored continuously - Regular back-ups of the server data will be made to GDPR-compliant cloud storage, and server disaster recovery measures are in place |
| Data server access | - Access to the admin panel has two-factor authentication enforced - Only a limited number of researchers will have access to the data server - There will be strict access permission rules applied per user role |
